# Potential Synergism between Novel Metal Complexes and Polymeric Brominated Flame Retardants in Polyamide 6.6

**DOI:** 10.3390/polym12071543

**Published:** 2020-07-13

**Authors:** Alistair F. Holdsworth, A. Richard Horrocks, Baljinder K. Kandola

**Affiliations:** 1 Institute for Materials Research and Innovation, University of Bolton, Deane Road, Bolton, Greater Manchester BL3 6HQ, UK; alistair.holdsworth@manchester.ac.uk (A.F.H.); B.Kandola@bolton.ac.uk (B.K.K.); 2 Now at School of Chemical Engineering and Analytical Sciences, University of Manchester, Oxford Road, Manchester M13 9PL, UK

**Keywords:** polyamide 6.6, tungstates, zinc stannate, synergism, bromine, flammability, thermal analysis, flame retardant, polymers, smoke, mechanistic determination

## Abstract

While environmental concerns have caused polymeric brominated primary flame retardants (PolyBrFRs) to be effective replacement monomeric species, few alternatives for antimony trioxide (ATO) have been developed beyond the zinc stannates (ZnSs). Previous research, which explored the interactions of aluminium (AlW), tin (II) (SnW) and zinc (ZnW) tungstates with several phosphorus-containing flame retardants in polyamide 6.6 (PA66), is extended to two PolyBrFRs: brominated polystyrene (BrPS), and poly(pentabromobenzyl acrylate) (BrPBz). On assessing the effect of each tungstate on the thermal degradation and flammability in combination with each PolyBrFR using TGA, UL94, LOI, cone calorimetry and TGA-FTIR, only ZnW and SnW showed significant increases in LOI (>26 vol.%). Both ZnW-BrPS- and ZnW-BrPBz-containing formulations yielded average UL94 ratings ≥ V-2 and TGA char residues (corrected for metals content at 500 °C) in air > 15 wt.%. BrPS-containing samples, especially those containing ZnW and SnW, generated peak heat release rates approximately 50% lower than the equivalent BrPBz samples. These reductions did not correlate with respective increases in LOI, suggesting that tungstate-PolyBrFR combinations influence pre-ignition differently to post-ignition behaviour. Calculated synergistic effectivities indicate that ZnW functions as a synergist in both pre- and post-ignition stages, especially with BrPS. TGA-FTIR and char analyses showed that, in addition to the vapour-phase activity normally associated with PolyBrFRs, condensed-phase processes occurred, especially for the ZnW-PolyBrFR combinations. Additionally, ZnW demonstrated significant smoke-suppressing properties comparable with zinc stannate (ZnS).

## 1. Introduction

The environmental pressures on halogen-containing and particularly bromine-containing flame retardants (BrFRs) began during the late 1980s, when concerns were raised about the formation of dioxins when polymers containing them were incinerated [1]. During the next 30 years the focus of concerns changed to their potential toxicological effects, particularly of polybrominated diphenyls and diphenyl ethers during the 1990s, leading to bans on the production and use of the former and similar restrictions of selected examples of the latter such as penta- and octa-bromo diphenylene ethers in 2005 [2,3]. Subsequently, and in spite of its widespread use and successful survival of risk assessments [4], decabromodiphenyl ether, because of evidence of its bioaccumulation and toxicity of its degradation products in the environment, has been similarly banned within both the USA [5] and EU [6]. However, in its place, polybrominated flame retardants such as brominated polystyrene and poly(pentabromobenzyl acrylate) are often used since their polymeric nature binds them into a polymeric or coating formulation, thereby reducing their ability to leach out into the surroundings [7] and so pollute the environment [8].

As the synergist antimony (III) oxide or trioxide (ATO or Sb_2_O_3_) is used in combination with the majority of halogen-based flame retardants, FRs, in use, attention has also recently focused upon its toxicity and potential carcinogenicity [9], although since about 1989 alternatives such as the low-toxicity zinc stannates (ZnSs) have been available, but at a higher price [10,11,12]. The zinc stannates also have the advantage of suppressing smoke, a further disadvantage of ATO, which tends to increase smoke formation. However, the ZnSs are not equally effective with all BrFRs and so may only be used with selected examples [12,13], though evidence of interactions with non-halogen-containing flame retardants such as those containing phosphorus are known [14].

Recent research by ourselves investigated over 150 metal complexes for their ability to promote char in polyamide 6.6 (PA66), chosen as a typical engineering polyamide of which zinc oxalate [15] was shown to interact positively with poly(pentabromobenzyl acrylate) in terms of reduction in cone calorimetric peak heat release rate (PHRR) and increased residual char levels. Subsequent work showed that aluminium (AlW), tin (II) (SnW) and zinc (ZnW) tungstates not only increased char formation and reduced PHRR values when present alone in PA66 [16], but when also in the presence of the phosphorus-containing FRs, aluminium diethyl phosphinate (AlPi) and AlPi in the presence of melamine polyphosphate, they increased their respective FR behaviours and, in the case of ZnW, reduced smoke formation [17].

The aim of this paper is to assess the interactions of these tungstates with the PolyBrFRs brominated polystyrene (BrPS) and poly(pentabromobenzyl acrylate) (BrPBz) when compounded in PA66 with respect to flammability performance, determination of potential mechanisms of action, and study of any potential synergism.

## 2. Experimental

### 2.1. Materials

**Figure 1:**
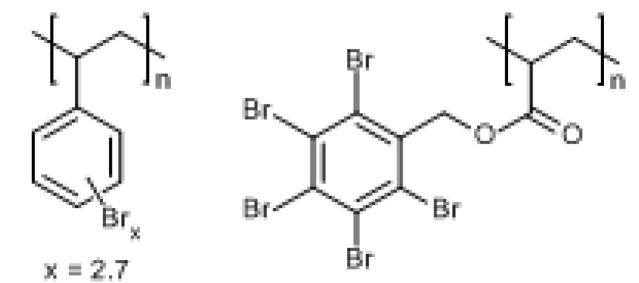
Structures of brominated polystyrene, BrPS (**left**) and poly(pentabromobenzyl acrylate), BrPBz (**right**).

The three tungstates (AlW, SnW and ZnW) were synthesised and characterised as previously reported [16]. These materials were calcined at 240 °C (under vacuum for SnW, to suppress oxidation of Sn (II) to Sn (IV)). Pure PA66 was acquired from Invista Engineering Polymers (compounding grade, 100% PA66, m.pt. 260 °C, MFI 19.56 g/min @ 280 °C), UK; brominated polystyrene (BrPS) and poly(pentabromobenzyl acrylate) (BrPBz), as commercial products FR803 (66 wt.% Br) and FR1025 (71 wt.% Br), respectively, were supplied by ICL-IP Ltd. These have the respective generic formulae as illustrated in Figure 1.

### 2.2. Polymer Composite Compounding

Compounding of all PA66 formulations has been described elsewhere, [16,17] but is repeated briefly here for convenience. This was undertaken using a laboratory-scale Thermo-Scientific twin-screw extruder with six barrel heating elements set progressively at 250, 255, 260, 265, 270 and 275 °C at a screw speed of 350 rpm. Prior to compounding, all PA66 polymer pellets and previously calcined flame retardant additive powders were dried at 80 °C for at least 36 h before processing.

Formulations outlined in Table 1 were selected based on the previous observations that 5 wt.% of each tungstate alone provided a marginal improvement in flame retardancy [16] and represented a typical level of inorganic synergist used commercially [18]. Additionally, compounding higher levels, especially AlW, proved to be difficult. Each PolyBrFR was present at 10 wt.%, a level that alone provided only a marginal increase in flame retardancy, but when in the presence of a typical synergist like ATO or ZnS would provide a possibly acceptable higher level [13].

Dry compounded pellets were pressed into plaques (170 × 170 × 3 mm) using a hot press at 260 °C with a pressure of 20 kg/cm^2^, followed by cutting into strips 12.7 mm wide for UL94 and LOI testing and 75 × 75 mm plaques for cone calorimetric analysis, where appropriate.

### 2.3. Fire Testing

Compounded PA66 samples were assessed for Limiting Oxygen Index (LOI) according to ASTM 2863, subjected to the UL-94 test in the vertical orientation, according to ISO 1210 and analysed by cone calorimetry at 50 kW/m^2^ heat flux (FTT cone calorimeter, Fire Testing Technology, UK) according to ISO 5660. Principal parameters determined included the peak heat release rate (PHHR), total heat release rate (THR) and total smoke release (TSR), with associated average errors of <10% [19].

### 2.4. Thermogravimetric and TGA-FTIR Analysis

Simultaneous thermogravimetric and differential thermal analyses (TGA/DTA) were conducted using a TA Instruments SDT 2960 analyser, with nominal sample masses of 10–12 mg and under a 100 mL/min flow of either air or nitrogen, using the method described elsewhere [15,17]. At least two experiments were undertaken for each sample. Transition temperatures were considered to be within ±1 °C. For TGA-FTIR studies, as also described in greater detail elsewhere [17,19], samples were heated from ambient to 600 °C at either 10 or 20 °C/min, the latter being used for the TGA-FTIR evolved gas analysis experiments. In the latter, the exhaust from the SDT 2960 thermal analytical module was connected to a heated gas cell (Thermo-Fisher Nicolet iS10) maintained at 250 °C mounted on a Thermo-Fisher Nicolet iS7 FTIR spectrometer via a heated, stainless steel gas line at 250 °C. Before each analysis, a 5 min isothermal equilibration stage at 100 °C was undertaken. FTIR data acquisition was started when the TGA/DTA ramp began with a gas transit time of 45 s from the exhaust of the TGA to the FTIR cell.

As described previously [17], the key evolved species, carbon dioxide, ammonia and aliphatic species including cyclopentanone that are possible fuels (designated CH_x_), were identified as being key indicators of significant mechanistic stages in the degradation of polyamide 6.6 [17,19,20], with respective IR absorption peaks monitored at 2357, 968 and 2933 cm^−1^. Absorption intensities were recorded over time and normalised with respect to unit sample mass after equilibration at 100 °C. Carbon dioxide profiles acquired under nitrogen and all others under air were corrected for atmospheric interference by the addition or subtraction of the lowest value of each spectral trace to give a zero baseline. The total amounts of CH_x_, NH_3_ and CO_2_ produced were calculated by summing all the FTIR intensity data points in arbitrary units.

### 2.5. Char Analysis

Selected chars from cone calorimetric studies were analysed by a Thermo-Scientific Nicolet iS10 FTIR instrument in combination with a diamond lens, attenuated total reflection (ATR) adapter. Detection of key metals within selected compounded chip samples and derived chars was undertaken by X-ray Fluorescence (XRF) experiments using the facilities of William Blythe Ltd., comprising a PANalytical Axios analyser and an Omnic software suite.

### 2.6. Synergistic Effectivity

The synergistic effectivities [21] were calculated using Equation (1), where Es is the synergistic effectivity, and X_p_, X_s_, X_fr_ and X_[fr+s]_ are the measured flammability parameters LOI and the percentage reduction in cone calorimetrically determined peak heat release rate (R_PHRR_). These represent the respective parameters for pure PA66, PA66 containing the synergist, PA66 containing the primary PolyBrFR and PA66 containing both.

Es = [X_[fr+s]_ − X_p_]/[(X_fr_ − X_p_) + (X_s_ − X_p_)] (1)

## 3. Results and Discussion

### 3.1. Thermogravimetric Behaviour

**Figure 2:**
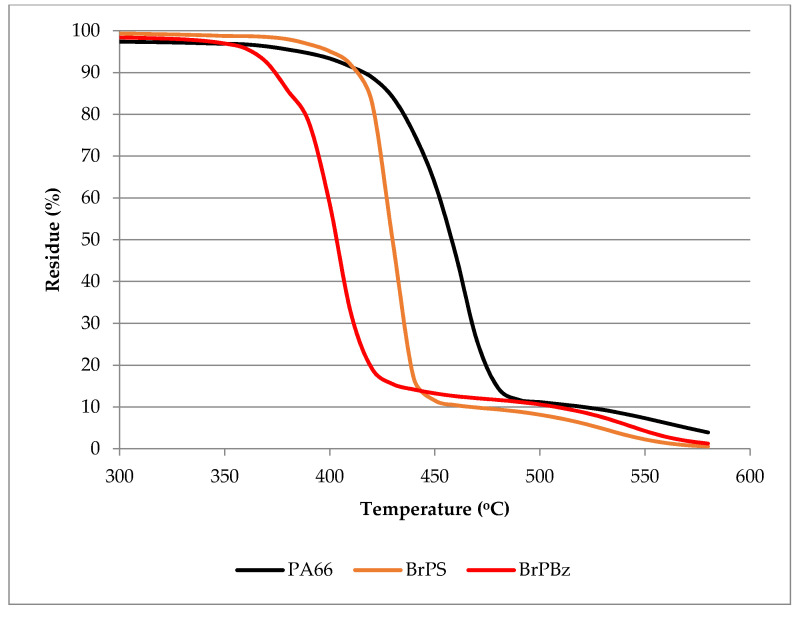
TGA mass losses of PA66 and PA66 samples containing 10 wt.% brominated polystyrene, BrPS, and and poly(pentabromobenzyl acrylate), BrPBz, under air.

Interactions between the three potential synergists (AlW, SnW and ZnW) and both selected PolyBrFRs (BrPS and BrPBz) were investigated based on the experimental matrix in Table 1, in which the brominated flame retardants were fixed at 10 wt.% total (equivalent to bromine levels in the range 6–7 wt.%) and the tungstates at 5 wt.% each. For both brominated flame retardants, selected levels were considered to not be sufficient to promote acceptably high degrees of flame retardancy on their own, so that any improvement in FR behaviour through the addition of the synergist would be clearly observed. Averaged TGA/DTA data under air is presented in Table 1 and the TGA curves from one set of TGA responses for each brominated flame retardant alone compounded in PA66 are shown in Figure 2. As can be seen, BrPBz significantly reduces the onset of degradation from ~386 to ~363 °C, while addition of BrPS shows a shift to a slightly higher temperatures, although subsequently showing a more rapid subsequent mass loss than either pure PA66 or BrPBz. It is evident that the presence of each PolyBrFR has significantly reduced the maximum rate loss temperatures relative to the control indicating significant interactions occurring with PA66. The effect of adding each tungstate to each PolyBrFR has little effect on the shapes of the respective TGA responses for the BrPS and BrPBz only formulations in Figure 2, with only slight shifts towards lower onset temperatures at 5% mass loss (T_5%_) and maximum volatilization rate temperatures (T_max_), as shown in Table 1.

Table 1 also shows that high temperature residue yields at 500 and 580 °C in air, while little affected by the presence of each PolyBrFR alone, show variable increases which appear to be tungstate dependent. These temperatures represent the levels of maximum oxidation-sensitised char formation and its subsequent oxidation respectively [17].

After correcting each R_500_ value by subtraction of an assumed 5 wt.% tungstate presence from each (although volatile bromides and oxybromides might result from PolyBrFR-tungstate interactions), residue levels at 500 °C significantly increase only when SnW and ZnW are present, with the addition of AlW appearing to have little or no char-enhancing effect. For these two former tungstates, it would appear that they are exerting some level of condensed-phase activity in the presence of each PolyBrFR.

### 3.2. Fire Performance

The UL94 and LOI fire performance data for PA66 with BrPS and BrPBz alone are presented in the upper part of Table 2, with respective values for each tungstate alone in PA66 inserted for comparison from our previous publication [16], where it was shown that their addition at 5 wt.% has a slight negative effect on the LOI with respect to PA66 with little if any effect on UL94 ratings. The generally poor reproducibility of the UL94 results is considered to be caused by less-than-ideal additive dispersion achieved when using a laboratory scale compounder. It was proposed that this slight lowering of the PA66 LOI from a base value of 22.6 vol.% is a consequence of changes in melt dripping behaviour caused by the previously reported char-forming effect, although the effect is not as evident in the presence of AlW. While there is minimal effect on LOI again by addition of BrPS and BrPBz alone, UL94 performance is improved and further inclusion of either SnW or ZnW increases LOI values significantly to >26.0 vol.% and improves the consistency of UL94 V-ratings with PA66-SnW-BrPS, PA66-ZnW-BrPS, and PA66-ZnW-BrPBz formulations yielding at least V-2 ratings.

Synergistic effectivity values cannot be sensibly calculated for the LOI data due to the negative denominators produced by SnW and ZnW, which have LOI values less than the control PA66 ((X_s_ − X_p_) see Equation (1)), leading to erroneously large negative Es values, and so in Table 2 we have assumed that (X_s_ − X_p_) = 0 and nominally given values >1 for SnW and ZnW-containing formulations since for the AlW-BrPS formulation which shows the low LOI value of 23.3, Es_(LOI)_ = 1.

**Figure 3:**
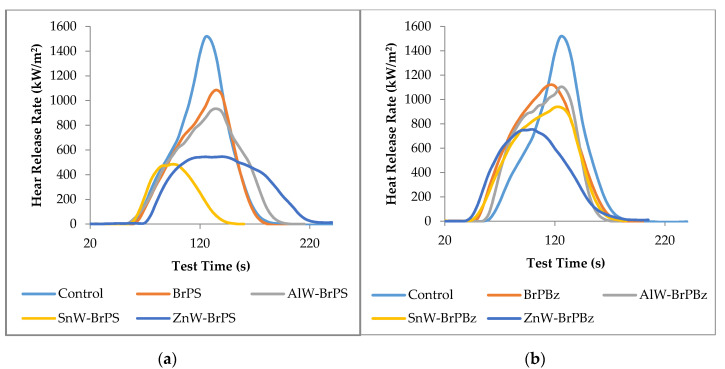
Cone calorimetry heat release rate curves for Table 1 samples. (**a**) formulations containing brominated polystyrene (BrPS) and (**b**) formulations containing poly(pentabromobenzyl acrylate) (BrPBz). AlW, SnW and ZnW = aluminium, tin (II) and zinc tungstates respectively.

The cone calorimetry heat release curves for this sample set are plotted in Figure 3. The behaviour of the cone calorimetric heat release rate curves resembles those reported previously by ourselves for tungstate/phosphorus FR formulations [17], in that times-to-ignition are reduced by the inclusion of both BrPS and BrPBz, corresponding to the reduced onset of degradation temperatures in TGA presented in Table 1. The associated PHRR, R_PHRR_ (the percentage reduction in PHRR), THR, and TSR values are also presented in Table 2, alongside synergistic effectivity values derived from R_PHRR_ values. It is clear that, generally, BrPS-containing samples, especially those containing ZnW and SnW, generate lower peak heat release rates, which are approximately 50% lower than the equivalent samples containing BrPBz. However, these respective reductions in PHRR values do not relate to the respective LOI and especially UL94 performances, as evidenced by the PA66-ZnW-BrPBz formulation that records both the highest LOI value (28.5 vol.%) and the highest V-ratings but only a moderate R_PHRR_ value. A similar non-correlation between changes in LOI and UL94 values and respective cone calorimetric data was noted in our previous study of the effect of phosphorus-containing retardant in PA66 [17]. This leads again to the conclusion that the effect of tungstate-PolyBrFR combinations influences pre-ignition-based parameters in a different manner to post-ignition parameters. This inference suggests also that the presences of ZnW and BrPBz have a greater condensed phase effect than other tungstate-PolyBrFR combinations, which is supported by the TGA data in Table 1 where the PA66-ZnW-BrPBz formulation is shown to have the highest R_500_ and R_580_ values.

The Es_(RPHRR)_ values listed in Table 2 indicate that SnW-PolyBrFR and ZnW-PolyBrFR formulations exhibit high levels of additivity, with ZnW-BrPS showing evidence of synergy (Es > 1). Therefore, it is proposed that zinc tungstate in particular might be a suitable synergist and alternative to ATO with some BrFRs. To test this hypothesis, the previously published results [14] regarding the relative effects of ATO and zinc stannate (ZnS) on BrPS and BrPBz are inserted into the lower part of Table 2 and respective Es_(LOI)_ and Es_(RPHRR)_ values calculated. The same problems arise with calculation of the former set of tungstate/PolyBrFR formulations with respect to LOI in that negative (X_s_ − X_p_) values are observed and so these imported samples are represented also by having values Es > 1 assuming (X_s_ − X_p_) = 0. 

**Figure 4:**
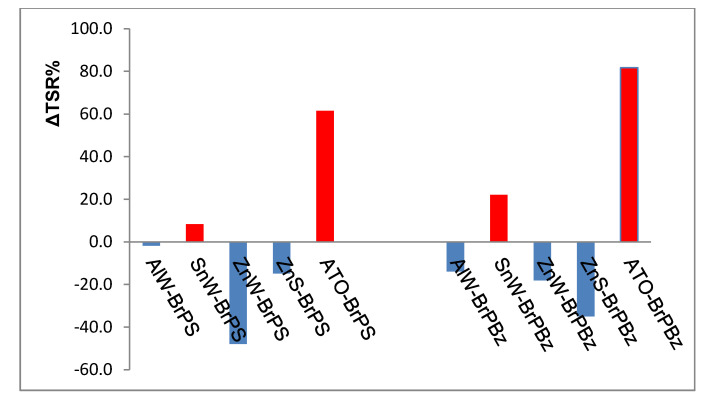
Percentage changes in smoke generation where the percentage change in total smoke release, ΔTSR% = [TSR_(MC/PolyBrFR)_/TSR_(PolyBrFR)_ − 1] × 100 and TSR_(MC/PolyBrFR)_ and TSR_(PolyBrFR_ are the respective smoke release values for each metal compound (MC) in combination with each PolyBrFR and PolyBrFR alone in PA66: Red columns indicate an increase and blue columns a decrease in smoke generation with respect to that from either brominated polystyrene, BrPS, or poly(pentabromobenzyl acrylate), BrPBz, present in PA66 alone. AlW, SnW and ZnW = aluminium, tin (II) and zinc tungstates respectively; ATO = antimony (III) oxide and ZnS = zinc stannate.

However, it is seen that all combinations have Es_(RPHRR)_ values either approaching unity for ATO-containing formulations and >1 for ZnS-containing formulations. By comparing these values with those for the respective SnW- and ZnW-containing formulations, it may be concluded that both tungstates are functioning in a synergistic capacity. With regard to smoke formation, while the addition of either PolyBrFR alone increases TSR values considerably with respect to PA66 as expected, the addition of each tungstate apart from SnW shows a general tendency for significant reductions. The smoke results plotted as percentage changes in TSR caused by addition of each tungstate, zinc stannate or ATO compared to the respective PA66-PolyBrFR sample (ΔTSR %) are presented in Figure 4 for each set of PolyBrFR combinations. Not surprisingly, the formulations containing ATO as the synergist show significant increases in smoke generation with only SnW behaving in a similar but much less severe manner. In contrast, the well-documented smoke suppressing property of zinc stannate, in the presence of each PolyBrFR is evident [12]. However, the greatest reduction in TSR is observed when ZnW is in the presence of either BrPS or BrPBz, which suggests that ZnW is comparable to zinc stannate as a smoke suppressant. It is interesting to note that the effectiveness of ZnW compared to ZnS with the two PolyBrFRs investigated is reversed, in that, ZnW is the more effective smoke suppressant with BrPS, while ZnS is the more effective with BrPBz.

### 3.3. TGA-FTIR Evolved Gas Analysis

The thermal degradation of polyamide 6.6 is more complex than that of the other aliphatic polyamides, primarily because chain scission combined with the elimination via ring formation of cyclopentanone, are precursors to cross-linking reactions [20]. In addition to the review by Schaffer et al. [20], we previously reviewed the thermal degradation mechanism of polyamide 6.6, including the majority of significant published work from the 1950–1960 period [17,19] from which the following volatile species identifiable by FTIR are used as mechanistic indicators.

Carbon dioxide is considered to be a product of polyamide chain scission, cyclopentanone and other fuel species, together designated as CH_x_, are evolved primary flammable volatiles and ammonia, produced primarily by condensation reactions, is an indicator of the formation of precursors to char-formation. These species were monitored during TGA using FTIR, namely CO_2_ at 2357 cm^−1^, aliphatic fuel formers (including cyclopentanone) CH_x_ at 2933 cm^−1^, and NH_3_ at 968 cm^−1^.

**Figure 5:**
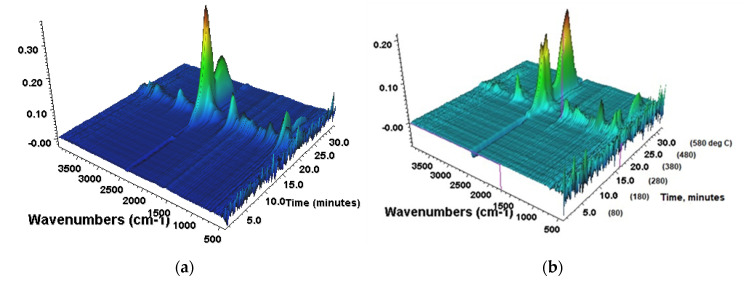
Exemplar 3D plots of TGA-FTIR data for (**a**) pure PA66 and (**b**) ZnW-BrPS under air.

Compounded specimens containing SnW and ZnW with associated controls were selected from the sample matrix presented in Table 1 and subjected to TGA-FTIR evolved gas analysis under both air and nitrogen. Samples containing AlW were omitted since these possessed the lowest flame retardancy from the results in Table 2. Exemplar FTIR spectra are shown in Figure 5 for the PA66 control and the ZnW-BrPS sample under air. In all spectra the peaks for CH_x_ (2933 cm^−1^), CO_2_ (2357 cm^−1^) and NH_3_ (968 cm^−1^) are significantly evident with no signs of either a carbon monoxide or CO doublet at 2100 and 2200 cm^−1^ or, in the PA66/BrPS (and PA66/PPBz) samples, HBr P and R IR bands at 2500 and 2600 cm^−1^, possibly due to reactions of HBr with the heated stainless steel gas line. Furthermore, it might be expected that other toxic species such as HCN (absorbing at ~2100 cm^−1^) would be produced in the presence of PolyBrFRs [22], and its absence here could also be a consequence of its reaction within the transfer line. The cyclopentanone C=O stretch absorption at 1750 cm^−1^ is also evident, though some overlap with other species present meant that this was not suitable for monitoring directly.

**Figure 6:**
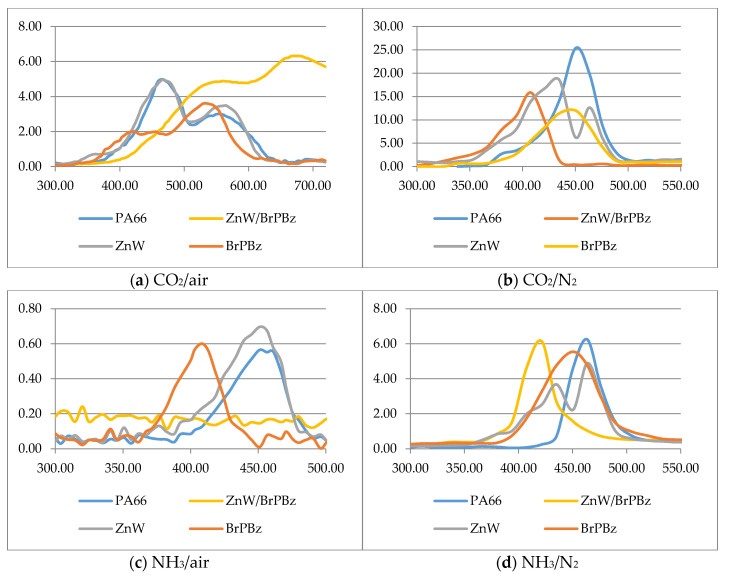

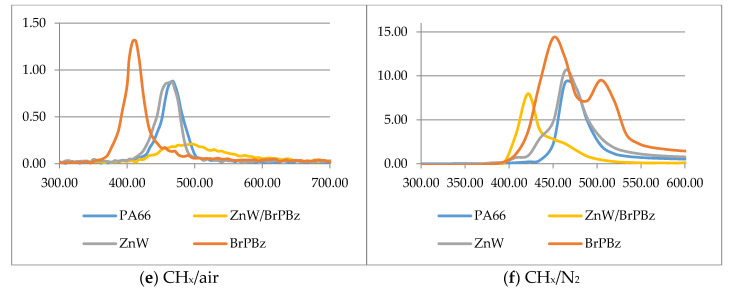
Plots of the intensity of CO_2_, NH_3_ and CH_x_ evolution for ZnW formulations with/without brominated polystyrene, BrPS, and poly(pentabromobenzyl acrylate), BrPBz, as measured by FTIR versus TGA temperature under air and nitrogen, respectively; CO_2_ (**a**,**b**), NH_3_ (**c**,**d**), CHx (**e**,**f**). Note that the temperatures here do not account for the approximate 45 s delay in recording the FTIR signal. SnW and ZnW = tin (II) and zinc tungstates respectively.

The CH_x_ (2933 cm^−1^), CO_2_ (2357 cm^−1^) and NH_3_ (968 cm^−1^) peak intensities as a function of temperature for all samples are shown in Figure 6. While the TGA responses in Figure 2 have typically shown that the thermal degradation of PA66 appears to be a single transition, the evolution of the different species occurring at different temperatures demonstrates the underlying complexity of the various reactions taking place [17,19,20,23]. Our recently published work regarding polyamide 6 thermal degradation showed that TGA responses for may in fact be resolved if high heating rates are used, thereby demonstrating that underlying reactions occur at differing rates [24].

**Figure 7:**
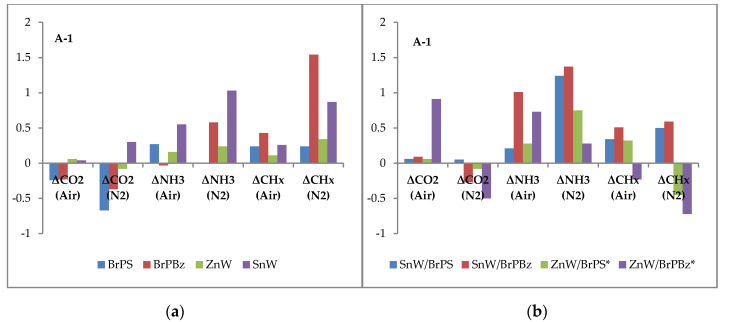
Variations in TGA-FTIR production of CO_2_, NH_3_, and CH_x_ by the samples listed in Table 3 relative to pure PA66 and plotted as (A-1.00, see Table 3) (**a**) BrPS, BrPBz, ZnW and SnW controls, (**b**) combinations of ZnW and SnW with BrPS and BrPBz in PA66. Negative values represent a reduction in gas/volatile production, while positive values represent increases relative to the control. BrPS = brominated polystyrene; BrPBz = poly(pentabromobenzyl acrylate); SnW and ZnW = tin (II) and zinc tungstates respectively.

The areas (A) under each of the respective gas/volatile evolution intensity versus temperature plots in Figure 6 were recorded and then normalised with respect to the respective PA66 control values under both nitrogen and air atmospheres, and results are presented in Table 3. These results are expressed with respect to the PA66 control (A = 1.00) as differential bar charts (A-1.00) in Figure 7. It should be noted that volatile emissions recorded above 580 °C, notably in Figure 6a for carbon dioxide emission from the ZnW-BrPBz formulation, is the ratio of the latter high residue (R_580_ = 20.8%—see Table 1) relative to the much lower value of the control (3.9%).

Referring to Figure 7, both PolyBrFRs alone reduce the CO_2_ production of PA66 under both air and nitrogen, which most likely reflects their ability to reduce volatile oxidation in the vapour phase. BrPS increases NH_3_ production under air, while BrPBz increases it under N_2_ and both PolyBrFRs and especially BrPBz increase the production of flammable volatiles (CH_x_) under both atmospheres. Combined with the other observations, this would suggest that any HBr formed during degradation of these compounds, although not observed for reasons stated above, would both alter the mechanism of and accelerate the degradation of PA66 [17], corresponding to the graphical data presented in Figure 2 and Table 1 and sensitisation of PA66 degradation by each PolyBrFR. The effects of inert and oxidising atmospheres on the subsequent degradation mechanisms of PA66 are complex, as numerous oxidation products behave somewhat differently than when heated under inert degradative conditions as evidenced by increased char formation, for example [13,17].

ZnW alone has relatively minimal effects on the degradation products of PA66 under either atmosphere, while SnW markedly increases both NH_3_ and CH_x_ production, especially under an inert atmosphere. The condensed-phase interactions of PA66 and its degradation products with inorganic additives often stem from Lewis-acid catalysis of these processes and there is evidence that SnW is more powerful than ZnW in this respect [17,25].

CO_2_ production is affected little by the combined SnW-PolyBrFR compositions under either atmosphere, though both NH_3_ and CH_x_ production are increased, which are especially notable in the SnW-BrPBz formulation in air. This formulation also has an inferior performance and R_PHRR_ value compared with the SnW-BrPS formulation, although char levels at 500 °C and LOI values are similar for both (Table 1 and Table 2).

The ZnW-PolyBrFR samples display differing behaviour depending upon which PolyBrFR is present. The ZnW-BrPS formulation relative to the PA66 control has minimal effect on CO_2_ production and increases NH_3_ release under both conditions and while increasing CH_x_ production under air, reduces it under N_2_. This moderate increase in CH_x_ and, hence, flammable volatile production in air is perhaps partly reflected in this sample’s increased LOI (26.2 vol%) value, although its high R_PHRR_ (70.5%) values in Table 2 provides evidence of a post-ignition, synergistic system (Es_(RPHRR)_ = 1.11). However, cyclopentanone is a significant component of the CH_x_ fuel fraction and this is known to promote cross-linking and eventual char formation, which could influence burning behaviour [19,20]. 

The ZnW-BrPBz composition diverges significantly from the control and samples containing either ZnW or BrPBz alone. CO_2_ production is increased under air and while reduced significantly under N_2_, is combined with an increase in NH_3_ evolution under both atmospheres and a substantial drop in production of flammable volatiles, especially under N_2_. The high R_500_ value (21.2%, Table 1) for this formulation and its subsequent combustion have been proposed as the cause of this increased CO_2_ production as noted above with reference to Figure 6a. The parallel increase in NH_3_ evolution may also be related to this high level of char formation, although the low R_PHRR_ value (45.5%, Table 2) and derived synergistic effectivity, Es_(RPHRR)_, of 0.84 suggests reduced post-ignition activity of the ZnW-BrPBz system compared with it superior pre-ignition activity in terms of the highest LOI (28.5 vol%) and >V-2 rating achieved of all the formulations studied.

From these results it can be concluded that, in addition to the vapour-phase activity normally associated with brominated flame retardants, further condensed-phase processes must also be occurring, especially for the ZnW-BrPBz sample. The high R_500_ and R_580_ values in Table 1 again confirm this inference.

The mechanism of smoke suppression produced by ZnW in the presence of BrPS or BrPBz remains poorly understood, although it could be attributed to the action of generated Lewis acids, such as ZnBr_2_ acting upon the precursor stages of smoke particle formation. In our related study of the effect of tungstates in combination with phosphorus-containing flame retardants in PA66 [17], while ZnW did not show any improvement in flame retardancy, it did show significant smoke suppressing properties, again associated with its probable Lewis acidic properties. It also is noteworthy that other recent work in our laboratories [26] has reported that zinc stannate in combination with BrPBz in PA66 does not show the expected significant vapour phase activity. It has previously been assumed that ZnS in combination with PolyBrFRs generally function by vapour phase mechanisms involving formation of volatile tin (II) bromide, tin (II) oxide and interactions between the latter and released bromine and hydrogen radicals [11,27]. However, this recent study has demonstrated that a considerable amount of bromine is trapped within the char that would otherwise be released into the vapour phase and that the interaction of bromine is primarily with zinc present in ZnS and not with tin.

### 3.4. Char Analyses

In addition to the TGA-FTIR analysis carried out as described above, char analysis of retained cone calorimetric residues was also undertaken using FTIR and XRF to allow for determination of the organic and inorganic components present. Several of the compositions tested by cone calorimetry left no residue at all, namely the PA66, BrPS and BrPBz controls and, as such, these could not be analysed.

#### 3.4.1. FTIR Analysis

**Figure 8:**
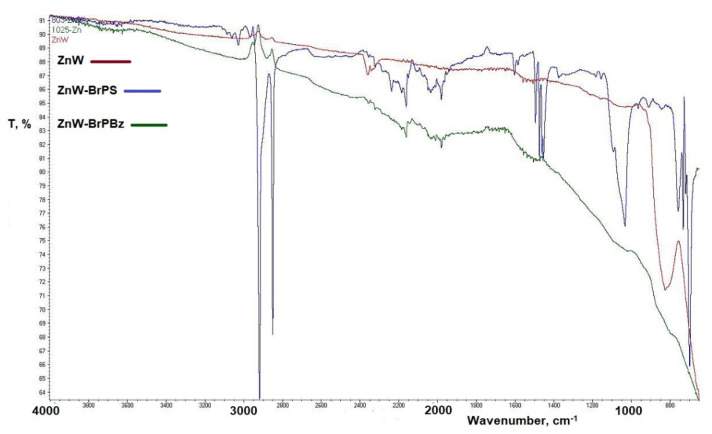
FTIR char spectra as percentage transmission (T%) derived from ZnW, ZnW-BrPS and ZnW-BrPBz formulation chars. ZnW = zinc tungstate; BrPS = brominated polystyrene; BrPBz = poly(pentabromobenzyl acrylate).

The char spectra are typified by those in Figure 8 derived from cone calorimetric chars from ZnW, ZnW-BrPS and ZnW-BrPBz formulations. These are generally characterised by carbon-hydrogen absorptions of a largely aromatic char structure, and spectra for the AlW, SnW and ZnW control chars contain few intense peaks, with only weak shoulder absorbances at 1200 cm^−1^. SnW in combination with either BrPS or BrPBz produces similar spectra, with strong absorbances observed at 2940 and 2850 cm^−1^, which correspond to aromatic C–H stretch modes. Aromatic ring-flexing peaks are present at 1500–1450 cm^−1^, although these are far sharper than the broad peaks observed for the phosphorus-containing samples previously reported [17]. While the observed spectrum for the ZnW-BrPS char is comparable to that of the SnW-PolyBrFR sample chars, the ZnW-BrPBz char spectrum is distinctly different from the other three bromine-containing formulations. A coherent char was produced by this sample noted previously from TGA studies (Table 2), and the FTIR spectrum of this in Figure 8 is devoid of intense peaks unlike the other formulations tested suggesting it is highly carbonaceous.

#### 3.4.2. XRF Analysis

Chars from all Br-containing compositions were examined by XRF analysis and results are presented as ratios between the key elements (i.e., M:W:Br and M:W:P, where M = Sn or Zn), as elements lighter than Na are not readily detected. Al could not be accurately measured as the chars were supported on aluminium foil. These results enabled comparison with the theoretical ratios calculated from the starting composition of each sample to determine which elements had been retained in the char, and which were lost to the vapour phase (see Table 4).

For all tungstate/PolyBrFR samples compositions tested (SnW-BrPS, SnW-BrPBz, ZnW-BrPS and ZnW-BrPBz), loss of bromine to the vapour phase is clearly evident, as shown by a clear reduction in the Br:W molar ratios. Losses of Zn and Sn are also observed with the former being significantly greater. However, the higher Br:W ratios for the ZnW-containing samples would suggest that more Br is retained in the condensed phase combination with the former than with SnW-containing formulations. This would suggest not only that respective metal/bromine losses to the volatile phase are not simply related, but also that condensed phase activity of ZnW is significant in its role as a synergist, especially with brominated polystyrene.

## 4. Conclusions

When present alone in PA66, both BrPS and BrPBz show little increases in char formation, as would be expected for these vapour phase active FRs, combined with their relatively low LOI values in the 22–23 vol.% range, and matched with UL94 ratings which average just less than V-2. In the presence of PolyBrFRs, only ZnW and SnW show significant increases in LOI (>26 vol.%), with both ZnW-BrPS and ZnW-BrPBz formulations yielding average UL94 ratings ≥ V-2 and corrected TGA char residues at 500 °C in air > 15 wt.% in the decreasing order ZnW > SnW >> AlW (see Table 1). This tungstate-PolyBrFR char-promoting effect is in the reverse order with respect to that for tungstate Lewis acidity [17,24], but corresponds to the respective decreasing boiling point order and thus volatility of the metal bromides in question, namely ZnBr_2_ (BPt. = 697 °C), SnBr_2_ (BPt. = 639 °C) and AlBr_3_ (BPt. = 255 °C). This mirrors the effects in our previous publication, where zinc stannate was demonstrated to have greater flame retarding efficacy than antimony trioxide (via SbBr_3_ formation, BPt. = 288 °C), most likely for a similar reason (see also Table 2) [13]. The possible effects of the poorly-characterised volatile tungsten oxybromides remain to be quantified, and would require the separate study of SnO, ZnO and WO_3_ in similar systems to those investigated here.

TGA-FTIR evolved gas data shows that both ZnW and SnW produce the greater changes in CO_2_, NH_3_ and CH_x_ profiles in air with respect to the controls containing each component alone. However and as noted above, the ZnW-BrPBz sample in air has evolved a significantly higher CO_2_ concentration relative to the SnW-BrPBz sample, most likely a consequence of elevated levels of char oxidation. This coupled with the reduced CH_x_ evolution could explain the superior flame retardancy of this latter sample where ignition and thermal resistance are the common underlying features of TGA, LOI and UL94 test procedures. In terms of reaction-to-fire behaviour and in particular comparison of PHRR values, the relative effects of SnW and ZnW show similarly higher percentage reductions when in combination with BrPS (R_PHRR_ = 67 and 71%, respectively, Table 2) than in combination with BrPBz (R_PHRR_ = 51 and 46%). Coupled with the cone calorimetric char analyses, which suggest that ZnW shows greater evidence of condensed phase activity than SnW, these figures also suggest that the effects of flame suppression by both SnW- and ZnS-PolyBrFR combinations are generally similar once ignition has occurred. 

To more fully understand the tungsten–bromine interactions involved would require further analysis beyond the scope of this present work. Suffice it to say that the effects of in-situ formation of and/or relative volatilities of possible intermediates and products, such as ZnO and SnO, and the volatile bromides mentioned above, plus the possible inclusion of SnBr_4_ (BPt. = 205 °C) and WBr_6_ (BPt. = 327 °C) and respective possible oxyhalides, may also be part of the explanation. Multicomponent analysis of the mixed-metal (M) tungstates (MWO_4_/M_2_(WO_4_)_3_) and the separate oxides (WO_3_, M_2_O_3_ and MO) would be required to determine further mechanistic intricacies of the interactions between PolyBrFRs and the compounds investigated here.

In summary, ZnW has demonstrated excellent smoke suppression properties, particularly in combination with BrPS, and this is most likely a consequence of its significant Lewis acidic, condensed phase activity, as evidenced by the char analytical results in particular. Clearly, this work has demonstrated that zinc tungstate acts both as a flame retardant synergist and smoke suppressant with both brominated polymers, BrPS and BrPBz. Whether or not these dual characteristics are observed when ZnW is in combination with other BrFRs requires further research in addition to further investigations into the mechanisms underlying its activity.

## Figures and Tables

**Table 1:** Thermogravimetric and differential thermogravimetric (TGA/DTG) results for PA66/tungstate/PolyBrFR formulations in air.

Sample	PA66	Br(BrPS)	Br(BrPBz)	MC*	TGA/DTG (Air)			
	Concentration, wt.%				T_5%_	T_max_, °C	R_500_, wt.% *	R_580_, wt.%
PA66	100.0				386	461	11.2	3.9
BrPS	90.0	10.0			400	430	8.1	0.5
BrPBz	90.0		10.0		363	402	10.6	1.2
AlW-BrPS	85.0	10.0		5.0	374	423	15.9 (10.9)	4.4
ZnW-BrPS	85.0	10.0		5.0	375	431	20.4 (15.4)	9.6
SnW-BrPS	85.0	10.0		5.0	368	424	20.9 (15.9)	10.3
AlW-BrPBz	85.0		10.0	5.0	367	405	15.1 (10.1)	4.6
ZnW-BrPBz	85.0		10.0	5.0	358	404	26.2 (21.2)	20.8
SnW-BrPBz	85.0		10.0	5.0	357	410	20.5 (15.5)	9.2

**Key:** BrPS = brominated polystyrene; BrPBz = poly(pentabromobenzyl acrylate); MC* indicates each tungstate; T_5%_ is the temperature to 5% mass loss, T_max_ is the DTG peak rate of mass loss, and R_500_ and R_580_ are the residues levels at those temperatures respectively (in °C); * bracketed values = (R_500_ − 5)%.

**Table 2:** Formulations, principal flammability parameters and derived synergistic effectivities for tungstate/zinc stannate/ATO-bromine-containing formulations in PA66.

Sample	Composition (%)			Flammability Parameters						
	PA66	MC *	PolyBrFR	UL-94 ***	LOI, Vol.%	Es_(LOI)_	PHRR, kW/m^2^	TSR m^2^/m^2^	R_PHRR_ %	Es_(RPHRR)_
PA66	100	-	-	F	22.6	-	1644	609	-	-
BrPS	90	-	10	V-2/V-2/F	22.9	-	1049	1821	36.2	-
BrPBz	90	-	10	V-2	22.3	-	1206	1447	26.6	-
AlW **	95	5	-	V-0/V-2/F	23.0	-	1156	927	29.7	-
SnW **	95	5	-	F/F/V-2	21.5	-	954	939	42.0	-
ZnW **	95	5	-	F	22.0	-	1190	638	27.6	-
AlW-BrPS	85	5	10	V-2/V-2/F	23.3	1	999	1789	39.2	0.60
AlW-BrPBz	85	5	10	F/F/V-2	22.3	0	1174	1246	28.6	0.51
SnW-BrPS	85	5	10	V-2	26.7	>1	546	1973	66.8	0.85
SnW-BrPBz	85	5	10	F/F/V-2	26.7	>1	802	1766	51.2	0.75
ZnW-BrPS	85	5	10	V-2	26.2	>1	485	949	70.5	1.11
ZnW-BrPBz	85	5	10	V-2/V-2/V-0	28.5	>1	896	1186	45.5	0.84
PA66 ****	100	-	-	F	24.5	-	1359	569	-	-
BrPS ****	85.1	-	14.9	V-2	23.8	-	1056	1730	-	-
BrPBz ****	85.9	-	14.1	V-2	23.9	-	990	1490	27.1	-
ZnS-BrPS ****	77.8	7.3	14.9	V-1	26.7	>1	354	1473	74.0	1.20
ATO-BrPS ****	79.0	6.1	14.9	V-2	31.0	>1	562	2794	58.6	0.88
ZnS-BrPBz ****	78.6	7.3	14.1	V-0	28.5	>1	163	969	88.0	1.32
ATO-BrPBz ****	79.8	6.1	14.1	V-2	31.9	>1	584	2707	57.0	0.80

Notes: BrPS = brominated polystyrene; BrPBz = poly(pentabromobenzyl acrylate); AlW, SnW and ZnW = aluminium, tin (II) and zinc tungstates respectively; * MC indicates each metal compound; ** values from [16]; *** a single rating indicates that all three test results achieved that value, otherwise individual test values given; F = fail; **** values reworked from [14], where PolyBrFR concentrations are equivalent to 10 wt.% Br; PHRR is peak heat release; TSR is total smoke release; R_PHRR_ % is the percentage reduction in peak heat release rate, PHRR, with respect to PA66; Es_(LOI)_ and Es_(RPHRR)_ are synergistic effectivities based on LOI and R_PHRR_ values respectively.

**Table 3:** TGA-FTIR gas/volatile absorption intensities (A) collected under air and N_2_, normalised to respective pure PA66 values (A = 1.00). The compositions of these samples correspond to those in Table 1 and Table 2. Those samples marked with an asterix (*) have the best fire performance.

Sample	CO_2_ (Air)	CO_2_ (N_2_)	NH_3_ (Air)	NH_3_ (N_2_)	CH_x_ (Air)	CH_x_ (N_2_)
Control	1.00	1.00	1.00	1.00	1.00	1.00
BrPS	0.76	0.33	1.27	1.01	1.24	1.24
BrPBz	0.77	0.63	0.97	1.58	1.43	2.54
ZnW	1.06	0.92	1.16	1.24	1.11	1.34
SnW	1.04	1.30	1.55	2.03	1.26	1.87
SnW-BrPS	1.06	1.05	1.21	2.24	1.34	1.50
SnW-BrPBz	1.09	0.73	2.01	2.37	1.51	1.59
ZnW-BrPS *	1.06	0.92	1.28	1.75	1.32	0.55
ZnW-BrPBz *	1.91	0.50	1.73	1.28	0.77	0.28

**Table 4:** Summary of the absolute molar ratios between heavy elements in PA66 plaques and char samples normalised with respect to tungsten present in all samples (W = 1.000). BrPS = brominated polystyrene; BrPBz = poly(pentabromobenzyl aacrylate); SnW and ZnW = tin (II) and zinc tungstates respectively.

Sample	Br:W	Sn/Zn:W
SnW/BrPS plaque	7.532	1.000
Char	0.040	0.673
SnW-BrPBz plaque	6.796	1.000
Char	0.053	0.638
ZnW-BrPS plaque	7.046	1.000
Char	0.368	0.284
ZnW-BrPBz plaque	3.842	1.000
Char	0.210	0.179
